# A Case of Endometrial Carcinosarcoma Containing Sertoliform Endometrioid Carcinoma Component

**DOI:** 10.1155/2021/5868818

**Published:** 2021-09-20

**Authors:** Satoru Munakata, Hanae Kushibiki, Taishi Akimoto, Tsuyoshi Yamashita, Norihiko Shimoyama

**Affiliations:** ^1^Department of Pathology, Hakodate Municipal Hospital, Hakodate, Hokkaido, Japan; ^2^Department of Obstetrics and Gynecology, Hakodate Municipal Hospital, Hakodate, Hokkaido, Japan

## Abstract

Carcinosarcomas (CSs) of the endometrium have admixture of malignant epithelial and mesenchymal components. The carcinomatous component exhibit endometrioid, serous, or clear cell differentiation, or are undifferentiated. CSs are considered homologous or heterologous according to the type of sarcomatous component. Sertoliform endometrioid carcinomas (SECs) of the endometrium which comprise a rare subtype of endometrial cancer, typically occur in the ovary. SECs as a carcinomatous component of CS of the endometrium have not been reported. Here, we report an endometrial carcinosarcoma that contains an SEC component. An 88-year-old female presented to a clinic with atypical genital bleeding. She was referred to our hospital and underwent total hysterectomy, bilateral adnexectomy and partial omentectomy due to endometrial carcinoma. Gross examination revealed a polypoid mass in the uterine cavity with massive myometrial invasion. Histologically, the tumor was a high-grade endometrioid carcinoma. In addition to an ordinary conventional endometrioid carcinoma, approximately 30% of the area exhibited sex cord-like pattern and contained small hollow tubules, anastomosing cords and trabeculae, and tightly packed nests. Immunohistochemically, the SEC component showed diffuse p53 staining. Sex cord-like area, especially the solid area, showed positive staining for EMA, vimentin, *α*-inhibin, CD99, calretinin, p53, CD56, synaptophysin, and chromogranin A, which is a staining pattern similar to that previously reported SEC of the endometrium. Diminished membranous and positive cytoplasmic staining for *β*-catenin was observed. This is the first case report of an endometrial carcinosarcoma containing an SEC component. SECs of the endometrium might exhibit sex cord-like differentiation in contrast to SECs of the ovary, which do not exhibit sex cord differentiation.

## 1. Introduction

Carcinosarcomas (CSs) of the endometrium contain an admixture of malignant epithelial and mesenchymal components. The carcinomatous components exhibit endometrioid, serous, or clear cell differentiation, or are undifferentiated. CSs are considered homologous or heterologous according to the type of sarcomatous component [1].

Sertoliform endometrioid carcinomas (SECs) of the endometrium comprise a rare subtype of endometrial cancer and typically refer to tumors in the ovary to avoid confusion from true sex cord-stromal tumors (SCSTs) [2–7]. Distinction of this type of the tumor is very important pathologically because the clinical behavior of SECs is very different from that of SCSTs. Recently, a similar histological type of endometrioid carcinoma was reported in the endometrium. To the best of our knowledge, only seven cases have been reported thus far [4–7]. Although negative immunohistochemical expression of *α*-inhibin has been reported in SECs of the ovary, one case in the endometrium was positively stained for *α*-inhibin [7]. To the best of our knowledge, SEC of the endometrium has not yet been reported as a carcinomatous component of CS. Here, we report a rare case of endometrial carcinosarcoma containing an SEC component, along with extensive immunohistochemical analysis including staining for *α*-inhibin.

## 2. Case Presentation

### 2.1. Patient

An 88-year-old female, G3P3, presented to a clinic with atypical genital bleeding. Her history included asthma and hypertension, but her family history was unremarkable. She was referred to our hospital due to enlarged uterus, and a tumor was found in the uterine cavity by ultrasonography. MRI revealed the uterine tumor with moderate density in T2WI. Myometrial invasion was also suspected. Endometrial cytology showed many papillary clusters of atypical cells, which suggested adenocarcinoma. In the endometrial biopsy specimen, many papillary nests of small atypical cells were observed. Immunohistochemically, cytokeratin AE1/AE3, vimentin, p53, CD56, synaptophysin, and chromogranin A were positive. However, ER, PgR, and WT-1 were negative. At this time, small cell neuroendocrine carcinoma was suspected. The patient then underwent total hysterectomy, bilateral adnexectomy and partial omentectomy. She did not receive any post-operative therapy, and is currently alive without any evidence of recurrence 4 months after surgery.

### 2.2. Tissue Processing and Immunohistochemistry

Tissue specimens were fixed in 10% buffered formalin. Paraffin embedded tissue was sectioned at a thickness of 3-4 *μ*m and was stained with hematoxylin and eosin. Immunohistochemistry was performed using the Bond Max® system (Leica Microsystems, K.K., Tokyo, Japan). The following antibodies were used and are listed in Table 1: Cytokeratin (AE1/AE3), EMA, PAX8, vimentin, estrogen receptor (ER), progesterone receptor (PgR), *α*-inhibin, CD99, calretinin, p53, WT-1, Melan-A, CD56, synaptophysin, chromogranin A, CD10, *α*-smooth muscle actin (SMA), myogenin, *β*-catenin, GATA3, MLH1, MSH2, MSH6, and PMS2. The immunohistochemistry results are presented in a semiquantitative manner as percentages of positively stained cells.

### 2.3. Gross and Histological Findings

**Figure 1:**
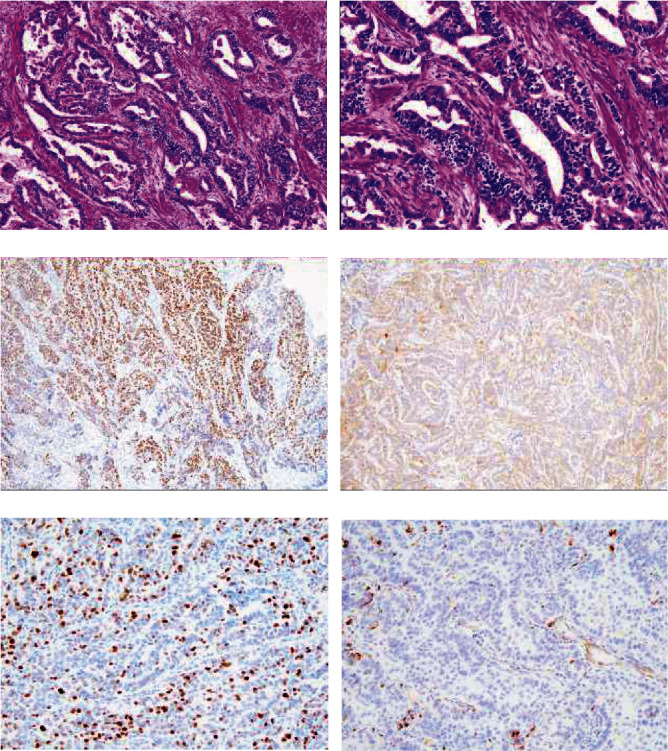
Histology image of the ordinary endometrioid carcinoma component. (a) Endometrioid glands were observed. (b) Higher magnification of the endometrioid carcinoma component. (c) PAX8 was positive. (d) p16 immunostaining was patchy. (e) p53 was wild type. (f) WT-1 was negative. (A, B: H&E staining, A: ×10, B: ×20, Immunostaining C, D: ×10, E,F: ×20).

**Figure 2:**
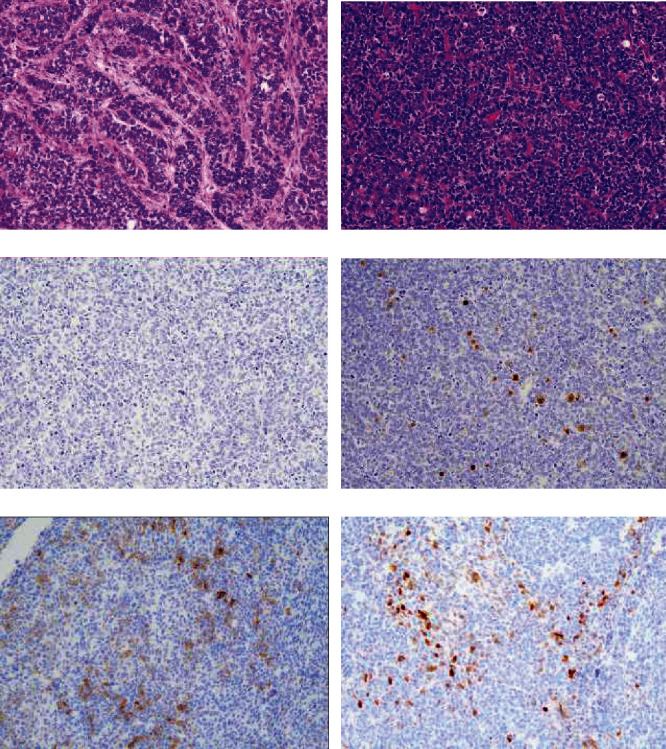
Histology image of the sertoliform endometrioid carcinoma component. (a) Sertoliform element in the glandular area. (b) Sertoliform element in the solid area. (c) AE1/AE3 was negative. (d) EMA staining was patchy. (e) *α*-inhibin showed positive staining. (f) Calretinin was also positive. (A, B: H&E staining, A, B: ×20, Immunostaining C-F: ×20).

**Figure 3:**
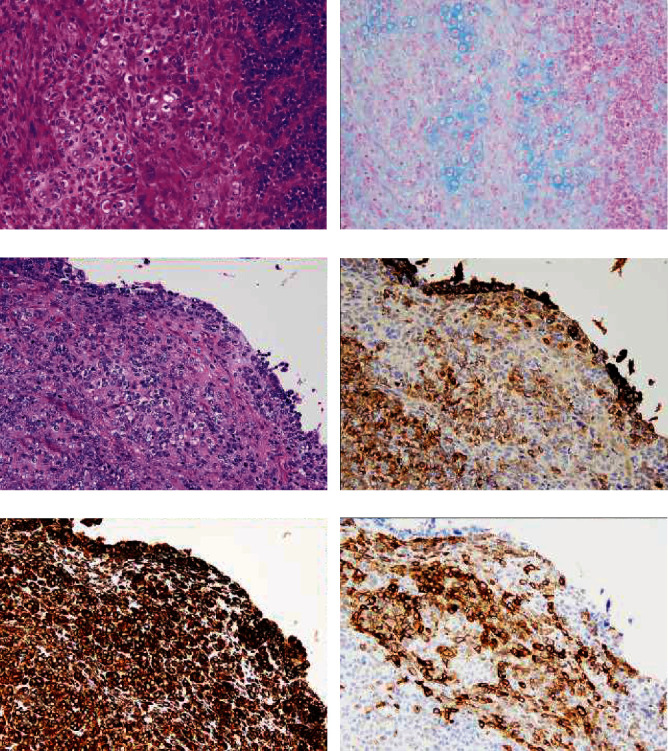
Histology image of the sarcomatous component. (a) Heterologous element with chondroid differentiation. (b) Chondroid stroma showed positive staining with Alcian blue. (c) Proliferation of spindle and polygonal stromal cells. (d) Epithelial cells were stained for AE1/AE3. (e) Vimentin was positive both in epithelial and stromal cells. (f) CD10 was positive in the sarcomatous cells. (A: H&E staining, ×20, B: Alcian blue ×20, Immunostaining C-F: ×20).

A large polypoid mass that measured 67 × 65 mm was observed inside the uterine cavity along with gross myometrial invasion. A uterine fibroid mass was also seen in the lower part of the uterine body. The omentum and bilateral adnexa were unremarkable. Histologically, the tumor was a high-grade endometrioid carcinoma that exhibited growth of irregularly shaped glands, which contained cells with eosinophilic cytoplasm and high-grade nuclei (Figures 1(a), (b)). These atypical glands of ordinary endometrioid carcinoma often showed a back-to-back appearance and cribriform features. Along with ordinary endometrioid carcinoma, approximately 30% of the area exhibited a sex cord-like pattern and contained a glandular area; this area contained small hollow tubules lined with columnar cells with apical cytoplasm, anastomosing cords, trabeculae, and tightly packed nests. Cells in the small hollow tubules had small, atypical nuclei with vesicular chromatin and pale cytoplasm (Figure 2(a)). The solid area contained small, atypical cells with dark oval vesicular nuclei and scant cytoplasm (Figure 2(b)). In some areas, atypical cell nests with chondroid differentiation (Figure 3(a)) and diffusely distributed spindle to polygonal atypical stromal cells were observed (Figure 3(c)). Deep myometrial invasion and lymphatic spread were also found. Histologically, the uterine fibroid mass was determined to be lipoleiomyoma. The omentum and the bilateral adnexa were not involved by the tumor.

### 2.4. Immunohistochemistry

**Figure 4:**
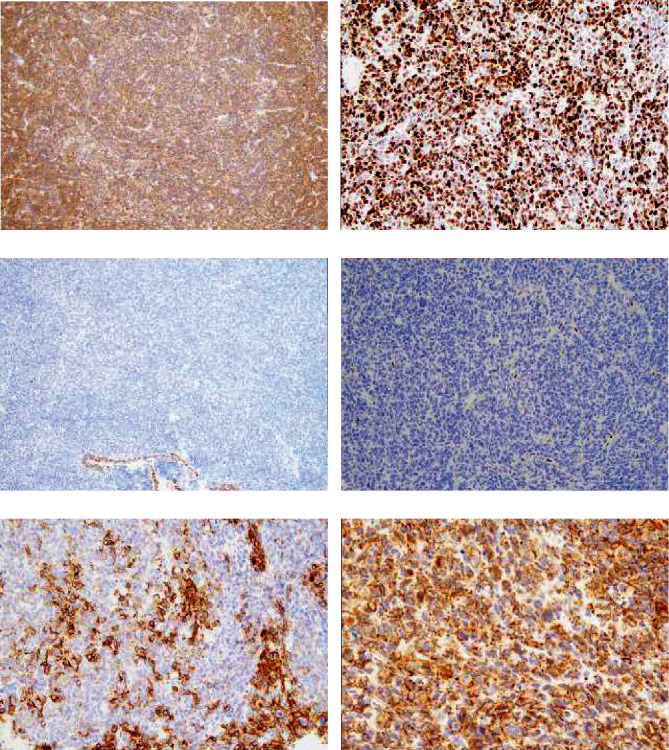
Immunohistochemistry image of the sertoliform endometrioid carcinoma component. (a) p16 showed diffuse staining in the solid area of the sertoliform element. (b) p53 also showed diffuse staining in the solid area. (c) PAX8 was negative. (d) ER was also negative. (e) CD56 was positive. (f) Membranous staining of *β*-catenin was diminished, while the cytoplasm was positively stained. (A, C: ×10, B, D, E: ×20, F: ×40).

The immunohistochemistry results were shown in Table 2. The ordinary endometrioid carcinoma element showed positive staining for cytokeratin (AE1/AE3), EMA, vimentin, ER, PAX8 (Figure 1(c)), and CD99. p16 staining was patchy (Figure 1(d)), and p53 staining was indicative of the wild-type gene (Figure 1(e)). WT-1 (Figure 1(f)) and GATA3 were both negative. The MIB1 labeling index was 21.9%. The mismatch repair gene products MLH1, MSH2, MSH6, and PMS2 showed positive staining. The glandular area of sex cord-like element showed positive staining for cytokeratin (AE1/AE3), EMA, vimentin, CD99, calretinin, CD56, synaptophysin, and chromogranin A, while p16 staining was patchy. Diffuse and strong staining indicated that p53 was mutated. The MIB1 labeling index was 34.4%. *β*-Catenin showed diminished membranous staining and positive cytoplasmic staining, which suggested aberrant expression. ER (Figure 3(d)), PgR, *α*-inhibin, PAX8 (Figure 3(c)), WT-1, Melan A, CD10, SMA, and myogenin were negative, while MLH1, MSH2, MSH6, and PMS2 were positive. Tightly packed solid nests of sex cord-like elements were positively stained for EMA (Figure 2(d)), vimentin, CD99, calretinin (Figure 2(f)), CD56 (Figure 4(e)), synaptophysin, and chromogranin A, which was similar to the glandular area. In contrast to the glandular area, *α*-inhibin (Figure 2(e)) was positive in the solid area. Diffuse staining for p16 (Figure 4(a)) and p53 (Figure 4(b)) was observed, and *β*-catenin showed diminished membranous staining and positive cytoplasmic staining (Figure 3(f)). MLH1, MSH2, MSH6, and PMS2 were all positive. The stroma that exhibited chondroid differentiation (Figure 3(a)) was stained with Alcian blue (Figure 3(b)). Atypical spindle and polygonal stromal cells (Figure 3(c)) were positive for vimentin (Figure 3(e)) and CD10 (Figure 3(f)), although those cells were negative for SMA and myogenin, which indicates endometrial stromal cell differentiation.

According to histological and immunohistochemical findings, our case was diagnosed with heterologous carcinosarcoma containing a sertoliform endometrioid carcinoma component.

## 3. Discussion

Endometrioid carcinoma mimicking a sex cord stromal tumor was first reported in the ovary as an SEC [2, 3]. Differentiation from SCSTs including Sertoli cell tumors and Sertoli-Leydig cell tumors is important pathologically because the prognosis of SECs is worse than that of SCSTs.

Immunohistochemically, SECs of the ovary have been reported to stain negatively for *α*-inhibin, which is useful for differential diagnosis between SECs and SCSTs [8–13]. SECs were also reported in the endometrium several years after reports of such tumors in the ovary [4–7]. Although SECs of the ovary have been reported to be negative for *α*-inhibin, Liang et al. found positive *α*-inhibin staining in such a tumor of the endometrium [7]. Similar to the case of Liang et al., our case also showed *α*-inhibin positivity in the sertoliform component. Our case also exhibited positive staining for calretinin and CD99, which are usually positive in SCSTs. According to this observation, the sertoliform pattern might exhibit sex cord-like differentiation in the endometrium.

Differential diagnoses of SECs in the endometrium include endometrial stromal sarcoma with sex cord-like elements (ESS-SCLEs), uterine tumors with sex cord-like elements (UTROSCTs), mesonephric adenocarcinoma (MNA), and corded and endometrioid carcinomas of the uterine corpus with sex cord-like formations, hyalinization, and other unusual morphologic features, which are also known as corded and hyalinized endometrioid carcinoma (CHEC).

ESS-SCLEs have components of endometrial stromal sarcoma along with sex cord-like elements [14]. These sex cord-like elements are usually positive for CD10 and occasionally stain for cytokeratin and *α*-inhibin, but they are almost always negative for EMA [15, 16]. In our case, endometrial stromal sarcoma components along with sex cord-like elements were observed. Although endometrial stromal sarcoma components showed positive immunoreactivity for CD10 (Figure 3(f)), the sex cord-like elements were positive for EMA and negative for CD10. In addition, our tumor contained typical endometrioid carcinoma components (Figures 1(a), (b)), and thus, it can be differentiated from ESS-SCLEs.

UTROSCTs were first described in 1976 [14]. Those tumors are located in the myometrium, and histologically, they contain areas of sex-cord differentiation, including plexiform cords, trabeculae, nests and well-formed tubules with lumina [14, 17]. Our tumor featured sex-cord differentiation but was primarily located in the endometrium. In addition, our tumor contained both conventional endometrioid carcinoma components and sarcomatous components. Immunohistochemically, UTROSCTs are positive for cytokeratin, vimentin, inhibin, CD99, Melan-A, CD56 and WT-1 [17]. Most tumors are negative for EMA, but one study reported weak to moderate staining [18]. Our tumor overlaps with UTROSCT in terms of positive staining for cytokeratin, vimentin, *α*-inhibin, CD99, and CD56 by immunohistochemistry, but our tumor was also positive for EMA and negative for Melan-A and WT-1. Therefore, our tumor can be differentiated from UTROSCTs. In the review by Baker and Oliva, UTROSCTs and ESS-SCLEs overlap immunohistochemically [16]. However, our tumor differs from both of those tumor types with respect to histological and immunohistochemical features.

MNA rarely arises from the uterine corpus and contains a variety of histologic components with features including tubulocystic, papillary, retiform, and solid growth patterns [19, 20]. Our tumor featured tubulocystic, papillary, and solid patterns, similar to what is observed in MNA. Immunohistochemically, MNA is usually positive for PAX8, GATA3, and CD10. Our case showed negative staining for PAX8, GATA3 and CD10 in the sertoliform element, and therefore, MNA was excluded.

CHECs were first described in 2005 [21]. CHECs are typical endometrioid carcinoma with frequent squamous differentiation, but they also contain epithelioid cells, spindle cells, or fusiform cells, which show a corded pattern and are embedded in hyalinized collagenous stroma. Some tumors form an osteoid pattern. Differential diagnoses of CHEC include carcinosarcoma and sertoliform endometrioid carcinoma. Our tumor partly resembles CHEC but did not contain hyalinized collagenous stroma. Immunohistochemically, CHECs show a very low frequency of p53 expression, in contrast to our tumor, which showed strong expression of p53. Therefore, our tumor can be differentiated from CHECs.

Although neuroendocrine markers were positive in the area of sertoliform differentiation, neuroendocrine markers are also known to be positive in sertoliform endometrioid carcinoma [13]. Morphologically it was difficult to exclude neuroendocrine carcinoma in our case, but positivity of sex cord markers, including *α*-inhibin and calretinin, by immunohistochemistry can exclude neuroendocrine carcinoma.

CSs predominantly affect postmenopausal women and appear as a polypoid mass in the uterine cavity [1]. This tumor contains an admixture of malignant epithelial and mesenchymal components with a diverse histological appearance. The carcinomatous components show endometrioid, serous, or clear cell differentiation, or are undifferentiated. The sarcomatous components consist of high-grade sarcoma, not otherwise specified (NOS), or heterologous elements, including rhabdomyosarcoma, chondrosarcoma, and osteosarcoma. To our knowledge, an SEC component has not been reported. The tumorigenesis of CSs has been debated. However, in recent studied, most of CSs have been reported to be clonal tumors [21, 22]. Moreover, most carcinosarcomas have *TP53* mutations [1, 22–24], and our tumor exhibited aberrant p53 expression mostly in the SEC element in accordance with the previous reports. Therefore, SEC components seem to be a part of the carcinosarcoma. Interestingly, in our case, the SEC components showed cytoplasmic *β*-catenin staining, and *β*-catenin protein expression abnormality have not been reported in SEC components. These observations imply that SEC component have a CTNNB1 gene mutation, although a molecular analysis was not performed in our case. The SEC components of our tumor showed *α*-inhibin, CD99, and calretinin positivity, which was concordant with the case reported by Liang et al. [7], while these markers are negative in SECs of the ovary. Therefore, SECs of the endometrium might exhibit sex cord-like differentiation along with epithelial differentiation. Both microscopic features and immunohistochemical results are important in the differentiation of SECs of the endometrium from other tumors with sex cord-like histology in the uterine corpus.

New molecular-based classification methods of endometrial carcinoma have recently been introduced by The Cancer Genome Atlas (TCGA) Research Network [25] and Talhouk et al. [26, 27]. Both methods classify endometrial cancer into four groups, which predict patient prognosis. According to those methods, endometrial cancers with p53 mutation (or aberrant p53 immunohistochemical expression) have the worst prognosis. Our case had aberrant a p53 mutation, and thus, intensive clinical care was mandatory. Although CSs have not yet been included in molecular-based classification systems, CSs should be included in those classification systems in the future.

In conclusion, we are the first to report a case of carcinosarcoma containing an SEC component, which exhibited *α*-inhibin and aberrant *β*-catenin expression. Our case supplemented current evidence of both sex cord-like differentiation and epithelial differentiation in SECs of the endometrium, which appear to be different from SECs of the ovary, although further investigation is necessary.

## Figures and Tables

**Table 1:** Primary antibodies used for immunohistochemistry.

Antibody	Clone	Host species	Source	Dilation	Antigen retrieval
Cytokeratin	AE1/AE3	Mouse	Nichirei	Ready-to-use	Protease
EMA	E29	Mouse	Nichirei	Ready-to-use	—
Vimentin	V9	Mouse	Nichirei	Ready-to-use	Heat
Estrogen receptor	SP1	Rabbit	Nichirei	Ready-to-use	Heat
Progesterone receptor	A9621A	Mouse	Nichirei	Ready-to-use	Heat
PAX8	BC12	Mouse	Nichirei	Ready-to-use	Heat
*α*-Inhibin	Clone R1	Mouse	DAKO	Ready-to-use	Heat
CD99	Cop58	Mouse	Novocastra	Ready-to-use	Heat
Calretinin	SP13	Rabbit	Nichirei	Ready-to-use	Heat
p53	DO-7	Mouse	Nichirei	Ready-to-use	Heat
WT-1	6F-H2	Mouse	Nichirei	Ready-to-use	Heat
Melan A	A103	Mouse	DAKO	1 : 50	Heat
CD56	1B6	Mouse	Novocastra	1 : 100	Heat
Synaptophysin	27G12	Mouse	Nichirei	Ready-to-use	Heat
Chromogranin A	Polyclonal	Rabbit	Nichirei	Ready-to-use	Heat
CD10	56C6	Mouse	Nichirei	Ready-to-use	Heat
*α*-Smooth muscle actin	1A4	Mouse	Nichirei	Ready-to-use	—
Myogenin	LO26	Mouse	Novocastra	1 : 50	Heat
*β*-Catenin	*β*-Catenin-1	Mouse	DAKO	Ready-to-use	Heat
GATA3	L50-823	Mouse	Nichirei	Ready-to-use	Heat
MLH1	ES05	Mouse	Novocastra	1 : 50	Heat
MSH2	ES05	Mouse	Novocastra	1 : 150	Heat
MSH6	PU29	Mouse	Novocastra	1 : 100	Heat
PMS2	M0R4G	Mouse	Novocastra	1 : 100	Heat

**Table 2:** Results of immunohistochemistry for each histological element.

Antibody	Sex cord-like element		Endometrioid element
	Glandular area	Solid area	
Cytokeratin (AE1/AE3)	70%	0%–	100%
EMA	80%	5%	100%
Vimentin	30%	100%	50%
Estrogen receptor	0%	0%	2%
Progesterone receptor	1%	0%	0%
*α*-Inhibin	1%	40%	0%
PAX8	0%	0%	95%
CD99	90%	95%	90%
Calretinin	20%	20%	20%
p16	50% (patchy)	95% (diffuse)	30% (patchy)
p53	70%	80%	40%
WT-1	0%	0%	0%
Melan A	0%	0%	0%
CD56	80%	60%	10%
Synaptophysin	5%	10%	0%
Chromogranin A	5%	1%	0%
CD10	0%	0%	0%
*α*-Smooth muscle actin	0%	0%	0%
Myogenin	0%	0%	0%
*β*-Catenin	Membranous, Cytoplasmic	Membranous, Cytoplasmic	Membranous
GATA3	5%	0%	0%
MLH1	95%	90%	50%
MSH2	90%	90%	90%
MSH6	70%	90%	60%
PMS2	80%	10% (weak)	30% (weak)
MIB1 labeling	34.4%	54.8%	21.9%
